# Investigating a potential association between agenesis of the third molars and variations in dental crown dimensions

**DOI:** 10.1371/journal.pone.0348605

**Published:** 2026-05-15

**Authors:** Gloria Guht, Christian Kirschneck, Nikolaos Daratsianos, Cristiano Miranda de Araujo, Natanael Henrique Ribeiro Mattos, Flares Baratto-Filho, Peter Proff, Diulia Pereira Bubna, Svenja Beisel-Memmert, Erika Calvano Küchler

**Affiliations:** 1 Department of Orthodontics, Medical Faculty, University Hospital Bonn, Bonn, Germany; 2 School of Dentistry, Medical Faculty, University of Tuiuti of Paraná, Curitiba, Brazil; 3 University of the Joinville Region, Joinville, Santa Catarina, Brazil; 4 Department of Orthodontics, University of Regensburg, Regensburg, Germany; 5 Department of Orthodontics, Goethe University Frankfurt, Carolinum, Frankfurt am Main, Germany; University of the Witwatersrand, SOUTH AFRICA

## Abstract

The aim of this study was to investigate the association between third molar agenesis and mesiodistal crown dimensions in individuals from Germany. The sample consisted of 314 (156 males and 158 females) orthodontic patients, divided into two groups: one group with individuals presenting at least one congenitally missing third molar and a control group with individuals having a full complement of 32 teeth. The mesiodistal crown width of each fully erupted permanent tooth was measured at its maximum dimension. The t-test was used to compare mesiodistal width between groups, with additional analyses according to gender and dental arch. A chi-square test evaluated the distribution of third molar agenesis among genders, and odds ratios (OR) with 95% confidence intervals (CI95%) were calculated. Pearson correlation assessed the relationship between the number of missing third molars and mesiodistal crown size. A total of 89 patients with third molar agenesis and 225 controls were included. Forty-three males and 46 females presented agenesis; with no significant association between gender and third molar agenesis (p = 0.760; OR = 1.08; 95% CI = 0.66–1.73). Mesiodistal dimensions were significantly different between males and females (p < 0.05). Patients with third molar agenesis exhibited a significant reduction (p < 0.05) in mesiodistal size across all teeth, most notably in second molars and maxillary lateral incisors. In males, all teeth in the agenesis group showed smaller dimensions compared to controls, with differences ranging from −0.43 to −0.15, the largest observed in the second maxillary molars (−0.43). In females, 18 of 28 teeth showed significant reductions, ranging from −0.42 to 0.00, with the largest difference in the left second mandibular molar (−0.42). The correlation between the number of missing third molar and mesiodistal size showed some negative moderate correlations, especially for lower canines (left r = −0.459, and right r = −0.534). In conclusion, third molar agenesis is associated with reduced mesiodistal crown dimensions in individuals from Germany, with this effect being more pronounced in males than in females.

## Introduction

Tooth agenesis is defined as the congenital absence of one or multiple teeth. It may occur in the primary or in the permanent dentition. Studies have shown that considering the global population the prevalence in the permanent dentition (6.4%) is higher than in the primary dentition (0.1%−0.2%) [1,2]. These studies often exclude the third molars, also called wisdom teeth. Including third molars the average prevalence of agenesis worldwide is 22.6%, with numbers varying greatly when limited to specific population groups [3]. After the third molars the second most common tooth to not be developed in the permanent dentition is the mandibular second premolar, followed by the lateral incisor of the maxilla and the second premolar of the maxilla [1]. The absence of up to six teeth is called hypodontia, while most of those affected are only missing one or two teeth (81.6%) [1]. The absence of more than six teeth (excluding third molars) is defined as oligodontia and the absence of the entire dentition as anodontia. Such a high prevalence, especially of the third molar, makes tooth agenesis one of the most common developmental dental anomalies. A meta-analysis showed that tooth agenesis is more likely to affect the female gender [1,4,5].

Among Europeans, 20–30% are congenitally missing at least one third molar [1,6]. Third molars are the last teeth to develop [7,8], and are the only teeth that develop completely after birth [9]. Furthermore, they are frequently affected by development alterations, for instance variation in tooth position/angulation, impaction and agenesis [7,10]. In general, dental anomalies including tooth agenesis are caused by an interplay of genetic, epigenetic and environmental factors during tooth development [1,11]. Recent studies have emphasized that specifically third molar agenesis is influenced by a strong genetic background [7,8,12]. Interestingly, genes involved in tooth agenesis have been also associated with reduced dental crown dimensions [13,14] suggesting that both traits share some genetic factors.

The association between tooth agenesis and dental crown dimensions has been investigated in a wide range of studies [15–17], however these studies often exclude the third molars. In the past five decades, few studies were performed and they suggested that patients with third molar agenesis had smaller teeth (mesiodistal dimension) compared to patients with a full complement of teeth [4,10,18–21]. Previous studies on the question were varied in their approach, with some focused only on specific teeth dimension, such as mandibular first molars [20] or in the non-metric analysis of maxillary lateral incisor [10,18]. However, the evidence connecting third molar agenesis and dental crown reduction is still weak due to the limited number of populations that have been previously investigated. Therefore, the aim of this study is to investigate the association between third molar agenesis and the mesiodistal crown dimensions of individuals from Germany.

## Materials and methods

### Sample

The sample consisted of 314 German orthodontic patients undergoing treatment at two universities located in Germany: The University of Regensburg and the University of Bonn. The local Ethics committees of University of Bonn (number: 2024–100-BO) and University of Regensburg (number: 19–1549–101) approved this study.

Patient records were screened and the data were collected from 20 May 2024 until 8 September 2025. The study was conducted in accordance with the ethical principles of the Declaration of Helsinki.

The patients evaluated were between 12.5 and 41 years old and comprised 156 males and 158 females. The patients were divided into two groups: one group consisting of patients congenitally missing at least one third molar, and the other group consisting of the control group. They were identified through the assessment of Orthopantomograms (OPG`s) that were taken after the age of 12.5. For the control group only patients with a full complement of teeth were chosen (32 teeth). Only individuals of European descent and patients in whom the presence or absence of agenesis was undoubtedly detectable were included. Exclusion criteria included syndromic patients or patients with illnesses that influence the tooth size or formation and patients with other congenitally missing teeth.

The sample size calculation was based on the expected mean difference in dental size between groups. The mean difference and standard deviation were derived from the results observed in a previous study in a European population from Slovenia [15]. We assumed a mean difference of 0.3 mm among groups and a standard deviation of ± 0.8 based on the average results observed [15]. The sample size was determined for independent-group design with a continuous primary endpoint using a two-sided independent samples t-test. An established alpha of 5% and power of 80% were used in the calculation and a case-to-control ratio of 1:2. Sample size was calculated using the ClinCalc online statistical tool [22], based on the standard formulas for the comparison of two independent means as described by Rosner [23]. A minimum sample of 84 patients with third molar agenesis and 168 controls would be required.

### Mesiodistal crown measurment

The software OnxyCeph^3TM^ LAB (Image Instruments, Chemnitz, Germany) was used to measure the mesiodistal crown width of each permanent tooth using 3D virtual models. If more than one dental scan was available, a dental scan from the beginning of the treatment was chosen. The measurements and the analysis of the OPG’s were made by one calibrated and trained dentist to limit variability. To determine the initial training and calibration, the examiner measured ten randomly selected digital scans from patients undergoing treatment in Bonn. Each scan was measured twice, with a two-week interval between the measurements. Intra-examiner reliability was calculated using the intraclass correlation coefficient (ICC), yielding 0.9993 (95% CI).

The mesiodistal tooth width was measured at the maximum width of every fully erupted permanent tooth, represented by the distance between the mesial and distal interproximal contact points of each tooth. The followed procedure was based on a protocol from previous publications [13]. In case of a rotated tooth or when the adjacent tooth was missing the point where the contact point would normally be chosen. Teeth with class I, II or III restorations, shape anomalies, interproximal caries, enamel reduction, fractured teeth and teeth with prosthetic restorations were excluded from the measurements.

### Statistical analysis

For the analysis of the data, GraphPad Prism software (Version 10.4.1) was used. The t-test was applied to determine whether the mesiodistal width differed significantly between the third molar agenesis group and the control group. Comparisons were also performed according to gender and dental arch (maxilla and mandible). The chi-square test was used to compare the distribution of third molar agenesis among groups, and odds ratios (OR) with 95% confidence intervals (CI 95%) were calculated. A Pearson correlation test was used to evaluate the strength and direction of the correlation between mesiodistal size and the number of missing third molars. For all statistical comparisons, an alpha level of 5% (p < 0.05) was adopted.

## Results

A total of 314 (156 males and 158 females) patients were analyzed, 89 patients with agenesis of at least one third molar and 225 controls. Forty-three males and 46 females presented third molar agenesis. Gender was not associated with third molar agenesis (p = 0.760; OR = 1.08, CI95% = 0.66–1.73). The sample description is presented in the Table 1.

**Table 1:** Sample characteristics according to the groups.

Variables	Controls	Third molar agenesis	p-value
*Gender*			
Males n (%)	113 (50.2)	43 (48.3)	0.760
Females n (%)	112 (49.8)	46 (51.7)	
*Age in years*			
Mean age (SD)	16.69 (5.8)	16 (5.4)	0.559
Minimum – Maximum	12.5 - 41	12.8 - 38	

Note: SD means standard deviation

Mesiodistal dimensions were statistically significantly different between males and females (p < 0.05, data not shown).

Table 2 presents the results of the mesiodistal size comparison among the control group and the third molar agenesis group. Patients with at least one congenitally absent third molar have a statistically significant reduction (p < 0.05) in mesiodistal size for each tooth in comparison to the patients in the control group. The teeth with the greatest reduction are in descending order the left second mandibular molar (−0.37), the left second maxillary molar (−0.35), the right second mandibular molar (−0.34) followed by the right maxillary second molar as well as the right and left maxillary lateral incisors (−0.32).

**Table 2:** Mesiodistal size comparison between control and third molar agenesis groups.

Tooth type	Controls				Third molar agenesis				Difference (mm)±SEM	P-value
	N	min-max (mm)	mean (mm)	SD (mm)	N	min-max (mm)	mean (mm)	SD (mm)		
***Maxillary***										
Right Second Molar	201	8.61-12.48	10.39	0.56	75	9.16-11.73	10.07	0.49	−0.32 ± 0.07	<0.0001
Right First Molar	220	9.75-12.47	10.87	0.53	85	9.24-11.92	10.64	0.46	−0.23 ± 0.07	0.0004
Right Second Premolar	221	6.16-8.55	7.15	0.37	86	5.78-7.72	6.94	0.37	−0.21 ± 0.05	<0.0001
Right First Premolar	222	6.25-8.58	7.40	0.37	83	6.35-8.22	7.22	0.35	−0.18 ± 0.05	0.0002
Right Canine	223	7.07-9.38	8.21	0.43	84	6.45-9.37	7.97	0.46	−0.24 ± 0.06	<0.0001
Right Lateral Incisor	225	5.97-8.83	7.18	0.54	88	5.70-8.27	6.86	0.53	−0.32 ± 0.07	<0.0001
Right Central Incisor	224	7.67-10.79	9.05	0.55	88	7.54-10.81	8.89	0.52	−0.16 ± 0.07	0.0216
Left Central Incisor	224	7.72-10.77	9.06	0.53	87	7.61-10.65	8.89	0.49	−0.17 ± 0.07	0.0093
Left Lateral Incisor	223	5.81-8.87	7.20	0.53	88	5.72-8.26	6.89	0.53	−0.32 ± 0.07	<0.0001
Left Canine	221	7.12-9.46	8.22	0.44	82	6.94-9.45	7.95	0.46	−0.27 ± 0.06	<0.0001
Left First Premolar	223	6.43-8.87	7.44	0.37	84	6.31-8.15	7.23	0.34	−0.21 ± 0.05	<0.0001
Left Second Premolar	223	6.42-8.21	7.14	0.36	83	5.82-7.68	6.97	0.37	−0.17 ± 0.05	0.0003
Left First Molar	221	9.74-12.53	10.94	0.52	86	9.36-11.90	10.65	0.47	−0.29 ± 0.06	<0.0001
Left Second Molar	193	8.91-12.68	10.35	0.58	76	8.85-11.28	10.00	0.47	−0.35 ± 0.07	<0.0001
***Mandibular***										
Left Second Molar	137	9.59-12.58	10.82	0.51	61	9.19-11.99	10.45	0.51	−0.37 ± 0.08	<0.0001
Left First Molar	222	10.00-13.13	11.47	0.60	88	10.02-12.56	11.23	0.50	−0.24 ± 0.07	0.0007
Left Second Premolar	223	6.35-8.99	7.62	0.44	87	5.99-8.67	7.44	0.44	−0.18 ± 0.06	0.0013
Left First Premolar	224	6.53-8.51	7.46	0.41	89	5.90-8.50	7.30	0.45	−0.16 ± 0.05	0.0025
Left Canine	225	6.01-8.42	7.09	0.43	88	6.04-8.05	6.88	0.40	−0.21 ± 0.05	0.0001
Left Lateral Incisor	224	5.42-7.43	6.26	0.38	89	5.49-7.52	6.10	0.36	−0.16 ± 0.05	0.0009
Left Central Incisor	224	4.40-6.66	5.66	0.35	89	4.63-6.54	5.54	0.32	−0.12 ± 0.04	0.0053
Right Central Incisor	225	4.58-6.74	5.66	0.35	89	4.75-6.61	5.55	0.31	−0.12 ± 0.04	0.0066
Right Lateral Incisor	224	5.42-7.20	6.25	0.37	88	5.26-7.37	6.10	0.35	−0.15 ± 0.05	0.0012
Right Canine	225	6.06-8.36	7.10	0.41	88	6.09-8.05	6.88	0.40	−0.22 ± 0.05	<0.0001
Right First Premolar	224	6.40-8.55	7.47	0.37	87	6.32-8.24	7.29	0.39	−0.18 ± 0.05	0.0002
Right Second Premolar	221	6.60-9.42	7.60	0.43	88	5.84-8.61	7.42	0.47	−0.18 ± 0.06	0.0012
Right First Molar	223	10.24-13.09	11.49	0.62	88	10.07-12.61	11.22	0.51	−0.27 ± 0.07	0.0003
Right Second Molar	129	9.82-12.59	10.78	0.52	57	9.43-11.26	10.44	0.40	−0.34 ± 0.08	<0.0001

Note: min means minimum; max means maximum; SD means standard deviation; SEM means standard error of the mean.

Once dental size presents sexual dimorphism, the analysis was also performed according to the gender, Table 2 presents the size comparison in males, and Table 3 represents the size comparison in females.

**Table 3:** Mesiodistal size comparison between control and third molar agenesis groups in males.

Tooth type	Controls				Third molar agenesis				Difference (mm)±SEM	P-value
	N	min-max (mm)	mean (mm)	SD (mm)	N	min-max (mm)	mean (mm)	SD (mm)		
***Maxillary***										
Right Second Molar	97	9.41-12.48	10.59	0.54	38	9.16-11.73	10.16	0.54	−0.43 ± 0.10	<0.0001
Right First Molar	111	9.75-12.47	10.98	0.56	42	9.49-11.92	10.68	0.49	−0.30 ± 0.10	0.003
Right Second Premolar	111	6.46-8.55	7.22	0.39	43	5.78-7.64	6.95	0.38	−0.28 ± 0.07	0.0001
Right First Premolar	112	6.47-8.55	7.49	0.37	40	6.38-8.22	7.31	0.35	−0.18 ± 0.07	0.0099
Right Canine	112	7.56-9.38	8.41	0.41	41	6.45-9.37	8.18	0.50	−0.23 ± 0.08	0.0034
Right Lateral Incisor	113	5.97-8.83	7.30	0.57	43	6.01-8.27	6.97	0.57	−0.33 ± 0.10	0.0013
Right Central Incisor	113	7.67-10.79	9.20	0.59	42	7.54-10.81	8.89	0.59	−0.31 ± 0.11	0.0037
Left Central Incisor	112	7.72-10.77	9.21	0.57	41	7.61-10.65	8.90	0.56	−0.32 ± 0.10	0.0028
Left Lateral Incisor	112	5.98-8.87	7.34	0.55	42	6.12-8.26	7.01	0.58	−0.33 ± 0.10	0.0014
Left Canine	111	7.32-9.46	8.41	0.45	40	6.97-9.45	8.16	0.46	−0.25 ± 0.08	0.0037
Left First Premolar	113	6.43-8.87	7.53	0.41	40	6.31-8.15	7.30	0.32	−0.23 ± 0.07	0.0014
Left Second Premolar	113	6.42-8.21	7.21	0.37	42	5.82-7.68	6.99	0.38	−0.23 ± 0.07	0.0009
Left First Molar	111	9.75-12.53	11.04	0.54	42	9.65-11.90	10.71	0.49	−0.34 ± 0.10	0.0006
Left Second Molar	93	8.91-12.68	10.54	0.60	37	8.85-11.28	10.11	0.53	−0.43 ± 0.11	0.0002
***Mandibular***										
Left Second Molar	67	9.83-12.58	10.94	0.53	31	9.19-11.99	10.60	0.55	−0.34 ± 0.12	0.004
Left First Molar	110	10.35-12.91	11.70	0.58	42	10.15-12.56	11.31	0.53	−0.39 ± 0.12	0.0002
Left Second Premolar	113	6.52-8.99	7.70	0.46	42	5.99-8.67	7.50	0.47	−0.20 ± 0.08	0.0175
Left First Premolar	113	6.56-8.51	7.54	0.42	43	5.90-8.50	7.39	0.45	−0.15 ± 0.08	0.0514
Left Canine	113	6.47-8.42	7.33	0.40	42	6.04-8.05	7.05	0.42	−0.27 ± 0.07	0.0002
Left Lateral Incisor	112	5.42-7.43	6.36	0.41	43	5.49-7.52	6.15	0.42	−0.21 ± 0.07	0.0058
Left Central Incisor	112	4.40-6.66	5.71	0.37	43	4.63-6.54	5.56	0.37	−0.15 ± 0.07	0.0225
Right Central Incisor	113	4.58-6.74	5.73	0.39	43	4.75-6.61	5.56	0.35	−0.17 ± 0.07	0.0149
Right Lateral Incisor	112	5.42-7.20	6.35	0.38	42	5.26-7.37	6.12	0.40	−0.23 ± 0.07	0.0012
Right Canine	113	6.42-8.36	7.32	0.38	42	6.21-8.05	7.06	0.41	−0.26 ± 0.07	0.0003
Right First Premolar	113	6.58-8.55	7.56	0.38	41	6.32-8.05	7.37	0.36	−0.19 ± 0.07	0.0072
Right Second Premolar	112	6.76-9.42	7.68	0.46	43	5.84-8.35	7.50	0.49	−0.19 ± 0.08	0.0277
Right First Molar	111	10.34-12.88	11.70	0.59	43	10.15-12.61	11.30	0.55	−0.40 ± 0.10	0.0002
Right Second Molar	69	9.82-12.59	10.90	0.57	29	9.70-11.26	10.55	0.39	−0.35 ± 0.12	0.003

Note: min means minimum; max means maximum; SD means standard deviation; SEM means standard error of the mean.

In males, all teeth in the third molar agenesis group exhibited statistically significant smaller dimensions compared to the male control group, with differences ranging between −0.43 and −0.15. The biggest difference was observed in the right and left second maxillary molars (−0.43) (Table 2).

In the female third molar agenesis group only 18 out of the 28 measured teeth displayed statistically significantly different dimensions compared to the female control group with differences ranging between −0.42 and 0.00. The biggest difference was observed for the left second mandibular molar (−0.42).

The comparison of the tooth reduction in the case of an agenesis of at least one maxillary third molar is represented in the S1 Table and the comparison of the tooth reduction in the case of an agenesis of at least one mandibular third molar is represented in the S2 Table.

The number of missing third molars ranged from 1 to 4 and the mean was 2.04 ± 1.19. The correlation between the number of missing third molars (in the third molar agenesis group) and mesiodistal size are presented in the Table 4. Negative moderate correlations were observed, especially for lower canines (left r = −0.459, and right r = −0.534). The correlation data is presented in the Table 4.

**Table 4:** Mesiodistal size comparison between control and third molar agenesis groups in females.

Tooth type	Controls				Third molar agenesis				Difference (mm)±SEM	P-value
	N	min-max (mm)	mean (mm)	SD (mm)	N	min-max (mm)	mean (mm)	SD (mm)		
***Maxillary***										
Right Second Molar	104	8.61-11.63	10.20	0.52	37	9.29-11.30	9.99	0.44	−0.22 ± 0.10	0.0256
Right First Molar	109	9.78-11.96	10.76	0.47	43	9.24-11.88	10.59	0.43	−0.17 ± 0.08	0.449
Right Second Premolar	110	6.16-7.86	7.08	0.33	43	6.08-7.72	6.94	0.37	−0.14 ± 0.06	0.0224
Right First Premolar	110	6.25-8.58	7.30	0.34	43	6.35-7.86	7.13	0.33	−0.17 ± 0.06	0.0066
Right Canine	111	7.07-8.91	8.00	0.36	43	7.05-8.37	7.78	0.33	−0.23 ± 0.06	0.0004
Right Lateral Incisor	112	5.99-8.34	7.06	0.48	45	5.70-7.79	6.75	0.48	−0.31 ± 0.08	0.0003
Right Central Incisor	111	7.76-10.05	8.89	0.46	46	7.85-10.12	8.90	0.45	0.00 ± 0.08	0.99
Left Central Incisor	112	7.72-10.08	8.91	0.44	46	7.97-9.94	8.89	0.41	−0.03 ± 0.08	0.7228
Left Lateral Incisor	111	5.81-8.40	7.07	0.48	46	5.72-7.84	6.78	0.45	−0.30 ± 0.08	0.0005
Left Canine	110	7.12-8.86	8.04	0.35	42	6.94-8.40	7.76	0.36	−0.28 ± 0.06	<0.0001
Left First Premolar	110	6.66-8.17	7.35	0.31	44	6.45-7.86	7.17	0.35	−0.19 ± 0.06	0.0014
Left Second Premolar	110	6.44-8.08	7.07	0.34	44	6.08-7.66	6.95	0.37	−0.12 ± 0.06	0.0671
Left First Molar	110	9.74-12.13	10.83	0.49	44	9.36-11.41	10.59	0.45	−0.24 ± 0.08	0.0059
Left Second Molar	100	9.05-11.41	10.17	0.50	39	9.30-10.82	9.90	0.39	−0.27 ± 0.09	0.0032
***Mandibular***										
Left Second Molar	70	9.59-11.51	10.71	0.46	30	9.45-11.08	10.29	0.42	−0.42 ± 0.10	<0.0001
Left First Molar	112	10.00-13.13	11.25	0.53	46	10.02-12.44	11.15	0.45	−0.10 ± 0.09	0.2753
Left Second Premolar	110	6.35-8.62	7.53	0.40	45	6.71-8.44	7.38	0.41	−0.15 ± 0.07	0.0319
Left First Premolar	111	6.53-8.28	7.39	0.38	46	6.33-8.40	7.22	0.43	−0.16 ± 0.07	0.0207
Left Canine	112	6.01-7.75	6.85	0.31	46	6.04-7.41	6.72	0.31	−0.12 ± 0.05	0.0265
Left Lateral Incisor	112	5.42-7.08	6.17	0.33	46	5.62-7.07	6.06	0.28	−0.11 ± 0.06	0.0598
Left Central Incisor	112	4.98-6.34	5.60	0.31	46	4.97-6.17	5.51	0.27	−0.08 ± 0.05	0.1113
Right Central Incisor	112	4.96-6.39	5.60	0.31	46	4.95-6.33	5.54	0.28	−0.07 ± 0.05	0.2038
Right Lateral Incisor	112	5.44-6.89	6.14	0.33	46	5.62-6.91	6.07	0.29	−0.07 ± 0.06	0.2219
Right Canine	112	6.06-7.59	6.88	0.30	46	6.09-7.36	6.73	0.31	−0.16 ± 0.05	0.0037
Right First Premolar	111	6.40-8.14	7.38	0.34	46	6.42-8.24	7.22	0.39	−0.16 ± 0.06	0.0114
Right Second Premolar	109	6.60-8.66	7.52	0.37	45	6.64-8.61	7.34	0.44	−0.18 ± 0.07	0.0126
Right First Molar	112	10.24-13.09	11.28	0.57	45	10.07-12.44	11.14	0.47	−0.14 ± 0.10	0.1457
Right Second Molar	60	9.90-11.61	10.65	0.42	28	9.43-10.98	10.34	0.39	−0.31 ± 0.09	0.0014

Note: min means minimum; max means maximum; SD means standard deviation; SEM means standard error of the mean.

## Discussion

Tooth agenesis is often associated with other developmental conditions, including craniofacial anomalies such as oral cleft, and other developmental dental anomalies such as microdontia/ peg shaped lateral incisors [24,25], and taurodontism [24] suggesting a shared underlying genetic basis. Other interesting relationship observed in cross-sectional studies are the association between tooth agenesis and mesiodistal tooth width reduction [11,15–17]. However, these studies often excluded the third molars [11,15]. A study from Keene in 1964 [20] investigated 195 male naval recruits and evaluated mandibular first molars. The author observed a crown reduction in the mandibular right first molar. A study from Lavelle et al. in 1970 [21] evaluated 301 casts from British subjects and observed an association between third molar agenesis and crown‌‌ dimension reduction (Table 5).

**Table 5:** Correlation between number of missing third molars and dental size.

Teeth	Maxillary		Mandibular	
	r	p-value	r	p-value
Right Second Molar	−0.291	0.079	−0.172	0.362
Right First Molar	−0.412	0.006	−0.339	0.021
Right Second Premolar	−0.164	0.293	−0.255	0.090
Right First Premolar	−0.239	0.121	−0.302	0.041
Right Canine	−0.355	0.019	−0.534	0.0001
Right Lateral Incisor	0.169	0.265	−0.387	0.008
Right Central Incisor	−0.115	0.446	−0.243	0.103
Left Central Incisor	−0.144	0.338	−0.265	0.074
Left Lateral Incisor	0.158	0.293	−0.367	0.012
Left Canine	−0.339	0.027	−0.459	0.001
Left First Premolar	−0.292	0.054	−0.392	0.007
Left Second Premolar	−0.250	0.114	−0.314	0.035
Left First Molar	−0.391	0.008	−0.437	0.002
Left Second Molar	−0.195	0.232	−0.402	0.034

Celikoglu et al. in 2011 [18] and Sujon in 2011 [10] investigated developmental dental anomalies associated with third molar agenesis in Turkish and Bangladesh patients (respectively). Both studies observed that these patients had higher prevalence of maxillary lateral incisor microdontia. Although microdontia was evaluated as a non-metric dental trait, these results support the hypothesis that third molar agenesis is associated with the reduction of crown size of the remaining teeth.

Thus, although some evidence suggests an association between third molar agenesis and dental crown size exist, this topic has been poorly explored in the literature and only few studies had focused in this type of teeth in the past 5 decades [10,18–21]. Therefore, the current study aimed to investigate if third molar agenesis is associated with crown width reduction in a German population in order to add new evidence in the literature. Our study confirmed previous findings and raises some new interesting concepts.

Besides the association between third molar agenesis and dental crown reduction, another interesting result observed here was the differences between males and females. Dental crown size exhibits sexual dimorphism, with males having larger crown dimensions than females. This difference is most pronounced in canines and molars [26]. In our study, we observed that the mesiodistal reduction occurred in all teeth of males with third molar agenesis, but this trend was not observed in females, in which only 18 teeth presented statistically significant reduction in the third molar agenesis group. Similar results were previously reported, Brook et al. [11] compared the amount of difference between the female and male patients, discovering that the reduction in female patients was less than the reduction in the male patients with hypodontia (excluding third molars) [11]. They stated that the higher reduction for the males could be explained by the fact that men have larger teeth in general and therefore have a higher reduction as well [11]. However, in our sample, some teeth did not show any or only a small morphometric change in females with third molar agenesis, suggesting that, in females, the factors involved concomitantly in agenesis and dental size do not play a role in some teeth, such as maxillary central incisor.

In our study, different types of teeth presented different levels of reduction. Brook et al. (2009) [11] observed that the teeth with the greatest reduction were the mandibular central incisor and maxillary lateral incisor. Furthermore, Fekonja (2013) [15] discovered in her study, that the teeth with the highest reduction are maxillary lateral incisors, lower central incisor and the lower second molar. Although Brook et al. (2009) [11] and Fekonja (2013) [15] included the general agenesis of permanent teeth and excluded the third molars, their results present some similarities to ours, and we observed bigger difference in molars and maxillary lateral incisors. Both of them concluded, that this result could suggest, that these teeth are the most vulnerable to disturbances throughout their development [11,15]. Interestingly, maxillary lateral incisors are frequently affected by agenesis and morphological alterations such as peg-shaped, dens invaginatus, talon cusp, and root alteration such as root angulation [27].

Other interesting observation that is important to highlight is the correlation observed between the number of missing third molars and crown dimensions of each permanent tooth. Although the correlation strength ranged from low to moderate, we were able to note that in some tooth types this moderate negative correlation exists, suggesting that patients with multiple third molar agenesis have smaller crown dimension. A similar result was also reported by Keene (1964) [20], which described a reduction more pronounced in subjects with four missing teeth. Garn and Lewis (1970) [4] concluded in their study on the American population with hypodontia, that this reduction is higher in multiple agenesis than in single agenesis. These suggest that the number of congenitally missing teeth are correlated with the crown size.

One of the strengths of the current study design is that only patients older than 12.5 years old were included. Third molars are the last teeth to develop and to erupt with eruption times ranging between 17 and 24 years [7,8]. Therefore, the agenesis of third molars cannot be reliably detected on panoramic radiographs before the patient has reached the age of 12.5 years [2]. This is because in some patients, before the age of 12 the tooth has often not started to mineralize [2]. For this reason, all cases of third molar agenesis were confirmed in radiographs in which the patients were 12.5 or older; to avoid the inclusion of individuals with delayed third molar development.

To understand the factors involved in mesiodistal crown dimensions of permanent teeth of patients with third molar agenesis is important for the planning of an orthodontic treatment, because it determines the spatial requirements. This knowledge regarding crown dimension in patients with third molar agenesis is also important for forensic estimation of gender based on dental size. Recognizing the association between third molar agenesis and reduced crown dimensions is critical in forensic odontometrics because this systemic reduction in tooth size can mimic female dental patterns, potentially leading to the misclassification of male remains if sexually dimorphic standards are applied without adjusting for known dental developmental anomalies.

While the current findings establish an interesting association between third molar agenesis and reduced mesiodistal crown dimensions, further cross-sectional research involving diverse ethnic cohorts is necessary to determine if these odontometric reductions are universal or to‌‌ population-specific.

## Conclusions

Third molar agenesis is associated with mesiodistal crown dimensions of individuals from Germany and this difference is more pronounced in males than in females. The number of missing third molars might also be correlated with the degree of reduction in mesiodistal crown width of the remaining permanent teeth.

## Supporting information

S1 Table Mesiodistal size comparison between control and third molar agenesis groups in the agenesis of maxillary third molars. (DOCX)

S2 Table Mesiodistal size comparison between control and third molar agenesis groups in the agenesis of mandibular third molars. (DOCX)
