# Supplementary material for: Investigating a potential association between agenesis of the third molars and variations in dental crown dimensions
Source: PLoS One. 2026 May 15;21(5):e0348605. doi: 10.1371/journal.pone.0348605 (PMC13178930; doi:10.1371/journal.pone.0348605)
Supplement: S1 Table — (DOCX) [file pone.0348605.s001.docx]

| Supplementary table 1. Mesiodistal size comparison between control and third molar agenesis groups in the agenesis of maxillary third molars. | | | | | | | | | | |
| --- | --- | --- | --- | --- | --- | --- | --- | --- | --- | --- |
| Tooth type | **Controls** | | | | **Third molar agenesis** | | | | Difference (mm)±SEM | P-value |
|  | N | min-max (mm) | mean (mm) | SD (mm) | N | min-max (mm) | mean (mm) | SD (mm) |  |  |
| ***Maxillary*** |  |  |  |  |  |  |  |  |  |  |
| Right Second Molar | 201 | 8.61-12.48 | 10.39 | 0.56 | 44 | 9.16-11.41 | 10.03 | 0.48 | -0.36±0.09 | 0.0001 |
| Right First Molar | 220 | 9.75-12.47 | 10.87 | 0.53 | 47 | 9.24-11.39 | 10.53 | 0.47 | -0.34±0.08 | <0.0001 |
| Right Second Premolar | 221 | 6.16-8.55 | 7.15 | 0.37 | 49 | 5.78-7.67 | 6.88 | 0.34 | -0.28±0.06 | <0.0001 |
| Right First Premolar | 222 | 6.25-8.58 | 7.40 | 0.37 | 47 | 6.35-8.09 | 7.13 | 0.35 | -0.26±0.06 | <0.0001 |
| Right Canine | 223 | 7.07-9.38 | 8.21 | 0.43 | 49 | 6.45-9.00 | 7.87 | 0.47 | -0.34±0.07 | <0.0001 |
| Right Lateral Incisor | 225 | 5.97-8.83 | 7.18 | 0.54 | 50 | 5.97-7.72 | 6.81 | 0.44 | -0.38±0.08 | <0.0001 |
| Right Central Incisor | 224 | 7.67-10.79 | 9.05 | 0.55 | 51 | 7.54-10.00 | 8.80 | 0.48 | -0.25±0.08 | 0.0031 |
| Left Central Incisor | 224 | 7.72-10.77 | 9.06 | 0.53 | 51 | 7.61-9.61 | 8.80 | 0.44 | -0.27±0.08 | 0.0009 |
| Left Lateral Incisor | 223 | 5.81-8.87 | 7.20 | 0.53 | 51 | 6.05-7.80 | 6.83 | 0.42 | -0.38±0.08 | <0.0001 |
| Left Canine | 221 | 7.12-9.46 | 8.22 | 0.44 | 48 | 6.94-8.71 | 7.86 | 0.46 | -0.36±0.07 | <0.0001 |
| Left First Premolar | 223 | 6.43-8.87 | 7.44 | 0.37 | 49 | 6.31-7.86 | 7.14 | 0.34 | -0.30±0.06 | <0.0001 |
| Left Second Premolar | 223 | 6.42-8.21 | 7.14 | 0.36 | 46 | 5.82-7.61 | 6.89 | 0.37 | -0.25±0.06 | <0.0001 |
| Left First Molar | 221 | 9.74-12.53 | 10.94 | 0.52 | 48 | 9.36-11.51 | 10.55 | 0.48 | -0.39±0.08 | <0.0001 |
| Left Second Molar | 193 | 8.91-12.68 | 10.35 | 0.58 | 45 | 8.85-9.94 | 11.28 | 0.58 | -0.41±0.09 | <0.0001 |
| ***Mandibular*** |  |  |  |  |  |  |  |  |  |  |
| Left Second Molar | 137 | 9.59-12.58 | 10.82 | 0.51 | 37 | 9.19-11.99 | 10.39 | 0.57 | -0.43±0.10 | <0.0001 |
| Left First Molar | 222 | 10.00-13.13 | 11.47 | 0.60 | 51 | 10.02-12.44 | 11.10 | 0.47 | -0.37±0.09 | <0.0001 |
| Left Second Premolar | 223 | 6.35-8.99 | 7.17 | 0.44 | 50 | 5.99-8.04 | 7.35 | 0.41 | -0.26±0.07 | 0.0001 |
| Left First Premolar | 224 | 6.53-8.51 | 7.46 | 0.41 | 51 | 5.90-7.95 | 7.21 | 0.41 | -0.25±0.06 | <0.0001 |
| Left Canine | 225 | 6.01-8.42 | 7.09 | 0.43 | 51 | 6.04-7.64 | 6.81 | 0.39 | -0.28±0.07 | <0.0001 |
| Left Lateral Incisor | 224 | 5.42-7.43 | 6.26 | 0.38 | 51 | 5.49-6.62 | 6.03 | 0.27 | -0.23±0.06 | <0.0001 |
| Left Central Incisor | 224 | 4.40-6.66 | 5.67 | 0.35 | 51 | 4.63-6.36 | 5.48 | 0.31 | -0.18±0.05 | 0.0009 |
| Right Central Incisor | 225 | 5.48-6.74 | 5.66 | 0.35 | 51 | 4.75-6.30 | 5.49 | 0.28 | -0.17±0.05 | 0.0013 |
| Right Lateral Incisor | 224 | 5.42-7.20 | 6.25 | 0.37 | 50 | 5.26-6.63 | 6.04 | 0.29 | -0.20±0.06 | 0.0003 |
| Right Canine | 225 | 6.06-8.36 | 7.10 | 0.41 | 51 | 6.09-7.85 | 6.81 | 0.39 | -0.29±0.06 | <0.0001 |
| Right First Premolar | 224 | 6.40-8.55 | 7.47 | 0.37 | 49 | 6.32-7.89 | 7.20 | 0.39 | -0.27±0.06 | <0.0001 |
| Right Second Premolar | 221 | 6.60-9.42 | 7.60 | 0.43 | 51 | 5.84-8.09 | 7.30 | 0.42 | -0.30±0.07 | <0.0001 |
| Right First Molar | 223 | 10.24-13.09 | 11.49 | 0.62 | 50 | 10.07-12.44 | 11.08 | 0.48 | -0.41±0.09 | <0.0001 |
| Right Second Molar | 129 | 9.82-12.59 | 10.78 | 0.52 | 43 | 9.70-11.26 | 10.49 | 0.40 | -0.44±0.10 | 0.0007 |
