# Supplementary material for: Investigating a potential association between agenesis of the third molars and variations in dental crown dimensions
Source: PLoS One. 2026 May 15;21(5):e0348605. doi: 10.1371/journal.pone.0348605 (PMC13178930; doi:10.1371/journal.pone.0348605)
Supplement: S2 Table — (DOCX) [file pone.0348605.s002.docx]

| Supplementary table 2. Mesiodistal size comparison between control and third molar agenesis groups in the agenesis of mandibular third molars. | | | | | | | | | | |
| --- | --- | --- | --- | --- | --- | --- | --- | --- | --- | --- |
| Tooth type | **Controls** | | | | **Third molar agenesis** | | | | Difference (mm)±SEM | P-value |
|  | N | min-max (mm) | mean (mm) | SD (mm) | N | min-max (mm) | mean (mm) | SD (mm) |  |  |
| ***Maxillary*** |  |  |  |  |  |  |  |  |  |  |
| Right Second Molar | 201 | 8.61-12.48 | 10.39 | 0.56 | 53 | 9.16-11.73 | 10.08 | 0.50 | -0.31±0.08 | 0.0003 |
| Right First Molar | 220 | 9.75-12.47 | 10.87 | 0.53 | 62 | 9.24-11.92 | 10.64 | 0.47 | -0.23±0.07 | 0.0022 |
| Right Second Premolar | 221 | 6.16-8.55 | 7.15 | 0.37 | 60 | 5.78-7.72 | 6.99 | 0.39 | -0.16±0.05 | 0.0029 |
| Right First Premolar | 222 | 6.25-8.58 | 7.40 | 0.37 | 59 | 6.35-8.22 | 7.26 | 0.37 | -0.13±0.05 | 0.0134 |
| Right Canine | 223 | 7.07-9.38 | 8.21 | 0.43 | 59 | 7.05-9.37 | 8.02 | 0.46 | -0.19±0.06 | 0.0039 |
| Right Lateral Incisor | 225 | 5.97-8.83 | 7.18 | 0.54 | 63 | 5.70-8.27 | 6.87 | 0.55 | -0.31±0.08 | <0.0001 |
| Right Central Incisor | 224 | 7.67-10.79 | 9.05 | 0.55 | 62 | 7.54-10.81 | 8.95 | 0.53 | -0.09±0.08 | 0.2263 |
| Left Central Incisor | 224 | 7.72-10.77 | 9.06 | 0.53 | 61 | 7.61-10.65 | 8.94 | 0.49 | -0.12±0.08 | 0.1176 |
| Left Lateral Incisor | 223 | 5.81-8.87 | 7.20 | 0.53 | 62 | 5.72-8.26 | 6.91 | 0.55 | -0.30±0.08 | 0.0002 |
| Left Canine | 221 | 7.12-9.46 | 8.22 | 0.44 | 57 | 7.12-9.45 | 8.00 | 0.46 | -0.23±0.07 | 0.0008 |
| Left First Premolar | 223 | 6.43-8.87 | 7.44 | 0.37 | 59 | 6.31-8.15 | 7.26 | 0.36 | -0.18±0.05 | 0.0011 |
| Left Second Premolar | 223 | 6.42-8.21 | 7.14 | 0.36 | 58 | 5.82-7.68 | 7.02 | 0.37 | -0.12±0.05 | 0.021 |
| Left First Molar | 221 | 9.74-12.53 | 10.94 | 0.52 | 62 | 9.36-11.90 | 10.66 | 0.49 | -0.28±0.07 | 0.0002 |
| Left Second Molar | 193 | 8.91-12.68 | 10.35 | 0.58 | 53 | 8.85-11.28 | 10.05 | 0.47 | -0.30±0.09 | 0.0008 |
| ***Mandibular*** |  |  |  |  |  |  |  |  |  |  |
| Left Second Molar | 137 | 9.59-12.58 | 10.82 | 0.51 | 45 | 9.45-11.99 | 10.48 | 0.49 | -0.34±0.09 | 0.0001 |
| Left First Molar | 222 | 10.00-13.13 | 11.47 | 0.60 | 62 | 10.02-12.56 | 11.25 | 0.52 | -0.22±0.08 | 0.0081 |
| Left Second Premolar | 223 | 6.35-8.99 | 7.16 | 0.44 | 61 | 5.99-8.67 | 7.47 | 0.48 | -0.14±0.06 | 0.0256 |
| Left First Premolar | 224 | 6.53-8.51 | 7.46 | 0.41 | 63 | 5.90-8.50 | 7.32 | 0.48 | -0.14±0.06 | 0.0216 |
| Left Canine | 225 | 6.01-8.42 | 7.09 | 0.43 | 62 | 6.04-8.05 | 6.88 | 0.43 | -0.29±0.07 | <0.0001 |
| Left Lateral Incisor | 224 | 5.42-7.43 | 6.26 | 0.38 | 63 | 5.49-7.52 | 6.12 | 0.39 | -0.14±0.05 | 0.0093 |
| Left Central Incisor | 224 | 4.40-6.66 | 5.66 | 0.35 | 63 | 4.63-6.54 | 5.53 | 0.33 | -0.12±0.05 | 0.013 |
| Right Central Incisor | 225 | 4.85-6.74 | 5.66 | 0.35 | 63 | 4.75-6.61 | 5.54 | 0.33 | -0.12±0.05 | 0.0148 |
| Right Lateral Incisor | 224 | 5.42-7.20 | 6.25 | 0.37 | 63 | 5.26-7.38 | 6.10 | 0.37 | -0.15±0.05 | 0.0062 |
| Right Canine | 225 | 6.06-8.36 | 7.10 | 0.41 | 62 | 6.09-8.05 | 6.89 | 0.42 | -0.21±0.06 | 0.0004 |
| Right First Premolar | 224 | 6.40-8.55 | 7.48 | 0.37 | 62 | 6.32-8.24 | 7.32 | 0.40 | -0.15±0.05 | 0.0054 |
| Right Second Premolar | 221 | 6.60-9.42 | 7.60 | 0.43 | 62 | 5.84-8.61 | 7.45 | 0.52 | -0.15±0.06 | 0.0216 |
| Right First Molar | 223 | 10.24-13.09 | 11.49 | 0.62 | 62 | 10.07-12.61 | 11.23 | 0.55 | -0.26±0.09 | 0.0029 |
| Right Second Molar | 129 | 9.82-12.59 | 10.78 | 0.52 | 43 | 9.70-11.26 | 10.49 | 0.40 | -0.30±0.09 | 0.0007 |
